# All roads lead to heterogeneity: The complex involvement of astrocytes and microglia in the pathogenesis of Alzheimer’s disease

**DOI:** 10.3389/fncel.2022.932572

**Published:** 2022-08-12

**Authors:** Marie-Kim St-Pierre, Jared VanderZwaag, Sophia Loewen, Marie-Ève Tremblay

**Affiliations:** ^1^Département de Médecine Moléculaire, Université Laval, Quebec City, QC, Canada; ^2^Axe Neurosciences, Center de Recherche du CHU de Québec, Université Laval, Quebec City, QC, Canada; ^3^Division of Medical Sciences, University of Victoria, Victoria, BC, Canada; ^4^Neuroscience Graduate Program, University of Victoria, Victoria, BC, Canada; ^5^Department of Biology, University of Victoria, Victoria, BC, Canada; ^6^Neurology and Neurosurgery Department, McGill University, Montréal, QC, Canada; ^7^Department of Biochemistry and Molecular Biology, University of British Columbia, Vancouver, BC, Canada; ^8^Center for Advanced Materials and Related Technology (CAMTEC), University of Victoria, Victoria, BC, Canada

**Keywords:** Alzheimer’s disease, heterogeneity, microglia, astrocyte, murine model, human brain samples

## Abstract

In recent years, glial cells have been acknowledged as key players in the pathogenesis of Alzheimer’s disease (AD), a neurodegenerative condition in which an accumulation of intracellular neurofibrillary tangles and extracellular fibrillar amyloid beta is notably observed in the central nervous system. Genome-wide association studies have shown, both in microglia and astrocytes, an increase in gene variants associated with a higher risk of developing late-onset AD. Microglia, the resident innate immune cells of the brain, and astrocytes, glial cells crucial for vascular integrity and neuronal support, both agglomerate near amyloid beta plaques and dystrophic neurites where they participate in the elimination of these harmful parenchymal elements. However, their role in AD pathogenesis has been challenging to resolve due to the highly heterogeneous nature of these cell populations, i.e., their molecular, morphological, and ultrastructural diversity, together with their ever-changing responsiveness and functions throughout the pathological course of AD. With the recent expansions in the field of glial heterogeneity through innovative advances in state-of-the-art microscopy and -omics techniques, novel concepts and questions arose, notably pertaining to how the diverse microglial and astrocytic states interact with each other and with the AD hallmarks, and how their concerted efforts/actions impact the progression of the disease. In this review, we discuss the recent advances and findings on the topic of glial heterogeneity, particularly focusing on the relationships of these cells with AD hallmarks (e.g., amyloid beta plaques, neurofibrillary tangles, synaptic loss, and dystrophic neurites) in murine models of AD pathology and post-mortem brain samples of patients with AD.

## Introduction

Alzheimer’s disease (AD), the most common form of dementia, is a neurodegenerative disease notably associated with severe synaptic loss and brain atrophy, clinically resulting in cognitive decline (Terry et al., 1991; Spires-Jones and Hyman, 2014; Pini et al., 2016; Halliday, 2017; Marino et al., 2019). The progressive impairment of learning and memory, among other cognitive functions, that characterizes AD is neuropathologically associated with intracellular neurofibrillary tangles composed of hyperphosphorylated tau protein and extracellular plaque deposition of amyloid beta (Aß) (DeTure and Dickson, 2019). Several key brain regions (e.g., hippocampus, dentate gyrus, entorhinal cortex, prefrontal cortex) involved in these functions are more vulnerable or prone to the various AD hallmarks aforementioned (Halliday, 2017; Mrdjen et al., 2019; Leng et al., 2021). However, while the presence of these abnormal features is a strong indicator of AD, the preserved cognition of some individuals with these AD hallmarks suggests that different mechanisms, apart from the pathological markers of AD, might be at play (Jack et al., 2016; Zolochevska and Taglialatela, 2016). Indeed, previous hypotheses regarding the pathogenesis of AD primarily comprise the amyloid cascade, which places the neurotoxic accumulation of the Aß protein at the center of the disease process (Hardy and Higgins, 1992; Ricciarelli and Fedele, 2017). Recent amendments to this hypothesis include mitochondrial dysfunction (Chakravorty et al., 2019), cellular senescence (Saez-Atienzar and Masliah, 2020; Walton et al., 2020; Guerrero et al., 2021) and vascular dysfunction (Govindpani et al., 2019). All these emerging hypotheses have highlighted the impact of glial cells (e.g., astrocytes, microglia) on AD pathology (Liu et al., 2018; Kaur et al., 2019; Hashemiaghdam and Mroczek, 2020).

Microglia first appear in the yolk sac where they egress to the central nervous system (CNS) around embryonic day 9.5 in mice (Ginhoux et al., 2010) and the 4–5th week of gestation in humans (Andjelkovic et al., 1998; Monier et al., 2006; Verney et al., 2010). From synaptic pruning and plasticity to surveillance of the parenchyma and removal of cellular debris, microglia exert crucial functions necessary for the proper development and maintenance of the CNS homeostasis throughout life (Davalos et al., 2005; Nimmerjahn et al., 2005; Paolicelli et al., 2011; Schafer et al., 2012). While understanding the role of microglia in the pathogenesis of AD has gained more traction in recent years with the advances in single-cell(sc)-omic techniques, it is still unclear if and/or how these cells can be modulated to help prevent and/or treat this neurodegenerative disease (Šimončičová et al., 2022). To study the mechanisms underlying this neurodegenerative disease, murine models of AD pathology, genetically altered to induce human AD hallmarks, are most commonly used (Jankowsky and Zheng, 2017). A summary of all the murine models of AD pathology mentioned in this Review is provided in Table 1. Studies from Sosna et al., and Spangenberg et al., have shown that long-term treatment starting early during the disease (at 1.5–2 months of age) with an inhibitor of colony-stimulating factor 1 receptor (CSF1R), notably crucial for microglial survival, was able to reduce the number of Aß plaques in the 5xFAD model [Swedish, Florida and London mutation in the amyloid precursor protein (APP) with M146L and L286V mutations in the humanized presenilin 1 (PSEN1) (Oakley et al., 2006)] (Sosna et al., 2018; Spangenberg et al., 2019). Oppositely, introducing a CSF1R inhibitor later on at 10 months of age did not detectably alter Aß load in the same 5xFAD model (Spangenberg et al., 2016) or in 12-month-old APP-PS1 mice [Swedish mutation in APP and humanized PSEN1 (Jankowsky et al., 2004; Radde et al., 2006)] (Unger et al., 2018), emphasizing the differential roles exerted by microglia across AD pathology progression (Casali et al., 2020). In addition, the functional spatio-temporal heterogeneity of microglia, observed even in non-pathological conditions and throughout the lifespan (Hammond et al., 2019; Zheng et al., 2021), confers an additional challenge alongside the diversity of their population comprised of different states [e.g., dark microglia, disease-associated microglia (DAM), neurodegenerative phenotype (MGnD)] observed in various mouse models of AD pathology and human post-mortem samples (Bisht et al., 2016b; Keren-Shaul et al., 2017; Krasemann et al., 2017).

**TABLE 1:** Mouse models of AD pathology used to investigate microglial and astrocytic heterogeneity.

Model	Mutations	Presence of Aß and/or neurofibrillary tangles (NFT)		References
		Aß	NFT	
5xFAD	APP [KM670/671NL Swedish, I716V Florida, V717I London] PSEN1 [M146L, L286V]	Yes	N/A	Oakley et al., 2006
APP-PS1	APP [KM670/671NL Swedish] PSEN1 [dE9]	Yes	No	Jankowsky et al., 2004
TgCRND8	APP [KM670/671NL Swedish, V717F Indiana]	Yes	No	Chishti et al., 2001
3xTg	APP [KM670/671NL Swedish] PSEN1 [M146V] MAPT [P301L]	Yes	Yes	Oddo et al., 2003
APP^NL–F–G^	APP [KM670/671NL Swedish, I716F Iberian, E693G Artic]	Yes	No	Saito et al., 2014
P301S	MAPT [P301S]	No	Yes	Allen et al., 2002; Bellucci et al., 2004; Yoshiyama et al., 2007
rTg4510	MAPT [P301L]	No	Yes	Ramsden et al., 2005; Santacruz et al., 2005
Thy-Tau22	MAPT [G272V, P301S]	No	Yes	Schindowski et al., 2006
JPNL3	MAPT [P301L]	No	Yes	Lin et al., 2003
TgF344 (rat)	APP [KM670/671NL Swedish] PSEN1 [dE9]	Yes	Yes	Cohen et al., 2013
Tg-SweArc	APP [KM670/671NL Swedish, E693G Arctic]	Yes	No	Lord et al., 2006

APP, amyloid precursor protein; MAPT, microtubule associated protein tau; PSEN1, presenilin 1.

In the last 5 years, the heterogeneous nature of astrocytes was also brought to the forefront of the dementia field with several studies underlining a myriad of astrocytic states in both health and disease (e.g., disease-associated astrocytes) (Liddelow et al., 2017; Habib et al., 2020; Escartin et al., 2021). Astrocytes were shown to play a key part in neuronal support; from sustaining metabolic needs by providing lactate to neurons according to the astrocyte-neuron lactate shuttle hypothesis, to recycling exocytotoxic glutamate into glutamine to be taken up by pre-synaptic axon terminals (Tsacopoulos and Magistretti, 1996; Pellerin et al., 2007; Verkhratsky and Nedergaard, 2018). In both patients with AD and mouse models of AD pathology, astrocytes were shown to interact with fibrillar Aß (Nagele et al., 2004) and dystrophic neurites found nearby Aß plaques (Gomez-Arboledas et al., 2018). For instance, ablation of astrocytes and proliferative astrocytes in organotypic 5xFAD brain culture slices and 9-month-old APP23/GFAP-TK mice [APP23 is a mouse model overexpressing a human APP with a Swedish mutation (Sturchler-Pierrat et al., 1997) crossed with glial fibrillary acidic protein (GFAP) thymidine kinase (TK) mice], respectively, both showed increased levels of Aß, indicating a crucial role for astrocytes in the resolution of AD pathology (Katsouri et al., 2020; Davis et al., 2021). Similar to microglia, astrocytes display a spatio-temporal heterogeneity during aging and in various pathological conditions (Lana et al., 2020). For instance, depending on their proximity to Aß plaques, astrocytes present varying morphologies (atrophy, hypertrophy) and molecular signatures in post-mortem brains of patients with AD and mouse models of AD pathology (Schitine et al., 2015). However, further investigations are required to fully understand the functional impact of their heterogeneous nature on the onset and progression of AD pathology.

Understanding the complex relationships of heterogeneous glial cells with their micro-environment will be crucial to unravel not only the mechanisms underlying AD, but also help to determine their responses to various potential therapeutic targets. Therefore, in this review, we will highlight the different microglial and astrocytic states observed in both mouse models of AD pathology and human post-mortem samples, discussing specifically how this heterogeneity affects their interaction with each other and the hallmarks of AD.

## Microglial heterogeneity in Alzheimer’s disease

Microglia aggregate near Aß plaques where they partially initiate and maintain a highly dynamic phagocytic and (neuro)inflammatory response (Manchikalapudi et al., 2019; Shukla et al., 2019; von Saucken et al., 2020; Grubman et al., 2021). While recent studies have helped elucidate the roles of microglia in the pathogenesis of AD, their functions throughout the course of the disease are still a topic boasting a variety of opinions. As much as microglia are able to take up Aß (Hickman et al., 2008; Sebastian Monasor et al., 2020), indicating their ability to initially inhibit plaque growth, they are ultimately not as effective at removing existing deposits later on in the disease process (Sebastian Monasor et al., 2020; Zhang G. et al., 2021). In addition, extensive literature highlights the impaired or altered functions that these cells exhibit throughout the progression of the disease (Prokop et al., 2013; Gyoneva et al., 2016; Kaur et al., 2019; Sebastian Monasor et al., 2020). How these changes in function may relate to morphological, ultrastructural, and transcriptomic states will be further discussed below.

### Morphological and ultrastructural diversity

Microglia survey the parenchyma through their ramified and highly motile processes. This morphological state, termed ramified or surveying microglia, can rapidly shift in response to micro-environmental changes (Davalos et al., 2005; Nimmerjahn et al., 2005). Ameboid microglia represent one of these morphological shifts and are observed notably after acute brain injury and in conditions where phagocytosis of debris or pathogens takes place (Giulian, 1987; Savage et al., 2019). This ameboid state has a large and round cell body which at first possess thin, almost invisible, processes that later on retract, which is thought to aid in microglia’s ability to migrate across the brain (Lu et al., 2011; Parakalan et al., 2012; Leyh et al., 2021). In AD pathology, the interaction between microglia and Aß can drive this morphological state. Curiously, morphological alterations linked to microglia’s close association with Aß plaques in the neocortex of 5-month-old male and female TgCRND8^wt/tg^; CXC3CR1^GFP/wt^ mice [mouse model with Swedish and Indiana mutations in APP (Chishti et al., 2001)] include a decrease in ramification, surface area, cell volume, number of processes and junctions (Plescher et al., 2018), an atrophy unlike the increased cell soma size traditionally associated with ameboid microglia (Leyh et al., 2021). This ameboid morphology has also been suggested to reflect microglial proliferation observed nearby Aß plaques in the post-mortem hippocampus of patients with AD (Marlatt et al., 2014).

Other morphological states include hypertrophic microglia–large cell bodies with short and thick processes–which have been identified in various pathological mouse models (e.g., traumatic brain injury, AD pathology), along with human AD cases (Walker and Lue, 2015; Zanier et al., 2015; Romero-Molina et al., 2018; Ohm et al., 2020). Indeed, this state is conserved in human post-mortem brain samples, being described in the hippocampus of aged individuals with AD (mean age of 77 and Braak stage IV-VI) (Bachstetter et al., 2015). Another state termed dystrophic microglia (also referred to as *senescent* in the literature) was uncovered in aging and notably near tau pathology (neurofibrillary degeneration) in AD human brains (Streit et al., 2004, 2009, 2020). These cells were additionally found in the middle temporal gyrus of healthy individuals (Swanson et al., 2020) and in the dorsal hippocampus of aged (7.5 years old) male tree shrews that developed hyperphosphorylated tau (Rodriguez-Callejas et al., 2020). Characteristic features of these cells include spherical swellings in their tortuous processes, notable accumulation of lipofuscin deposits (Streit et al., 2004, 2009, 2020; Lopes et al., 2008), and immunohistoreactivity to L-ferritin, a marker of cellular senescence (Lopes et al., 2008; Swanson et al., 2020). However, recent studies suggest that this microglial morphology could be linked to the pH of the brain rather than AD pathology as it was shown that lowering the pH of the brain was associated with an increased abundance of dystrophic microglia (Paasila et al., 2019). While hypoxia is associated with low pH, this study investigated the effect of changing pH without altering the oxygen content (Paasila et al., 2019).

Ultrastructural heterogeneity, including microglia’s diverse relationships with Aß and dystrophic neurites, can be observed in AD pathology using electron microscopy (EM) (Stalder et al., 1999; El Hajj et al., 2019). EM is a powerful imaging tool allowing to demystify the presence and functional state of various organelles within cells and their interactions with the micro-environment, but also the topographical heterogeneity of microglia. For instance, using scanning EM, Dyne et al., investigated the ultrastructural morphology (cell body shape, processes length, surface topology, aspect ratio) of human C20 cells [immortalized human microglial cell line from resected adult human brain samples] that were treated for 24 h with Aß. These cells showed a diverse topography based on their surface (designated as smooth, blebbed, ruffled or pitted depending on the number and size of their cavities) and displayed enlarged pores compared to an anti-inflammatory condition [interleukin (IL)-4 treatment for 24 h], a change hypothesized to be associated with phagocytosis (Dyne et al., 2021).

EM imaging was also employed to investigate ultrastructural microglial alterations in 14-month-old APP-PS1 male mice where microglia were shown to possess increased markers of cellular stress [e.g., endoplasmic reticulum (ER) dilation], a phenomenon which was exacerbated in microglia associated with dystrophic neurites and Aß (El Hajj et al., 2019). Microglial ultrastructural heterogeneity in AD pathology is also highlighted by the presence of dark microglia, a state associated with numerous markers of cellular stress and advanced/tertiary lysosomal organelles (Bisht et al., 2016b; St-Pierre et al., 2019, 2020). While this state is rarely present in adulthood during normal physiological conditions, it is notably more abundant in pathological conditions, including in 14-month-old APP-PS1 male mice (Bisht et al., 2016b). There are notable differences between dark microglia and their non-dark (or more typical) counterparts, which includes markers of oxidative stress (e.g., a condensed, electron-dense cyto- and nucleoplasm, altered mitochondria, dilated ER) and a remodeled nuclear chromatin pattern (Bisht et al., 2016a,b; St-Pierre et al., 2019, 2020). Dark microglia possess highly ramified, dark processes which frequently interact with axon terminals and dendritic spines (Bisht et al., 2016a); suggesting that they likely play a role in the synaptic dysfunction and loss observed in AD.

### Microglial signature heterogeneity

Genome-wide association studies uncovered numerous genes associated with a higher risk of developing late-onset AD [reviewed in Bertram and Tanzi (2009)]. One of these genes, triggering receptor expressed on myeloid cells 2 (*trem2)*, was shown to be crucial for the appearance of various microglial states, including the DAM reported in male and female 5xFAD mice at 3, 6, and 8 months of age (Keren-Shaul et al., 2017). The DAM transcriptomic state is associated with a TREM2-dependent decrease of homeostatic genes (e.g., *p2ry12, tmem119*) and increase of disease signature genes (e.g., *clec7a, spp1, itgax*) (Keren-Shaul et al., 2017). The necessity of TREM2 for the emergence of DAM was also confirmed by independent studies in which 7-month-old male and female 5xFAD mice were crossed with either homozygous or heterozygous TREM2-/- mice and injected intracerebrally with tau. The latter resulted in a reduced expression of DAM genes in the 5xFAD × TREM2+/- mice compared to 5xFAD × TREM2+/- mice, which was further exacerbated by the whole-brain knock-out of TREM2, when examined in both the cortex and hippocampus of these mice (Delizannis et al., 2021).

**FIGURE 1:**
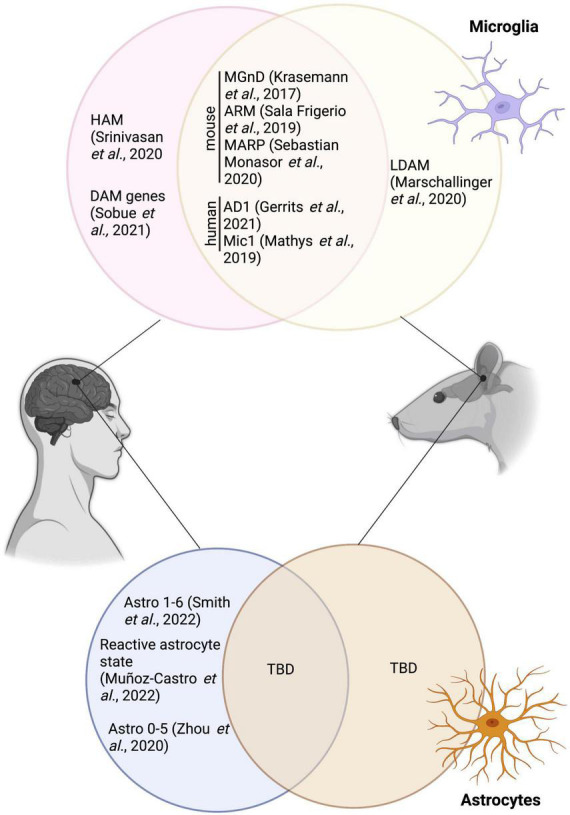
Overlap between the various microglial and astrocytic signatures described in human and mice, and the disease associated microglia and disease-associated astrocytes. HAM, human AD microglia; MGnD, neurodegenerative disease phenotype; ARM, activated-response microglia; MARP, microglial Aß response proteins; AD1, Alzheimer’s disease 1; LDAM, lipid droplet-accumulating microglia; TBD, to be determined. Figure was created using Biorender.

DAM were suggested to restrict themselves/reside primarily nearby Aß plaques–making it an important area of interest for therapeutical targeting (Keren-Shaul et al., 2017). Since the initial discovery by Keren-Shaul et al., this particular microglial signature has been uncovered in several other mouse models of AD pathology, including in 9-month-old homozygous APP-KI male and female mice [intercrossing of the heterogenous APP^NL–F–G^-KI mice, a mouse model with a Swedish, Iberian and Artic mutation in APP (Saito et al., 2014)] (Swartzlander et al., 2018). A summary of the overlap between DAM and other microglial states mentioned in this review is provided in Figure 1. In addition, DAM genes were observed notably in 6-month-old male TgCRND8 mice using RNA sequencing on laser capture microdissected non- and plaque-associated microglia; the DAM signature was largely restricted to nearby Aß plaques, highlighting the necessity of Aß plaques to induce this unique state (Rothman et al., 2018). Of note, while primary mouse microglial cells treated 12 h with fibrillar Aß recapitulated the DAM signature, cells treated for 12 h with oligomeric Aß did not, stressing the importance of an amyloid conformation for this particular microglial response (McFarland et al., 2021). Tau, in conjuncture with Aß, was also shown to drive the expression of the DAM signature in 7-month-old 5xFAD × P301S mice injected stereotactically with tau [P301S is mouse model expressing microtubule associated protein tau (MAPT) with a human PS301 mutation (Allen et al., 2002; Bellucci et al., 2004; Yoshiyama et al., 2007)] (Lodder et al., 2021) and 13-month-old APP-PS1 female mice both with and without the P301L mutation that induces the production of hyperphosphorylated tau (Natunen et al., 2020). An increased expression of DAM genes (e.g., *clec7a*, *cd68*, *trem2*) was also shown in tau pathology mouse models; e.g., in the hippocampus of 9–12-month-old P301S male and female mice (Romero-Molina et al., 2018) and in the cerebral cortex of 7-month-old rTg4510 mice [model expressing P301L mutation in MAPT (Ramsden et al., 2005; Santacruz et al., 2005)] (Sobue et al., 2021).

Further investigation identified various substates of DAM emphasizing the vast heterogeneity observed even within individual microglial states. These include, for instance, a pro- or anti-inflammatory substate in 6–8 month-old female 5xFAD mice (Rangaraju et al., 2018), and a senescent substate (as shown by telomere shortening, increased beta-galactosidase activity and changes in the expression of genes such as *serpine1*, *cdkn1/2a* and *casp8* associated with senescence) in 4, 6, 10 and 12/13 month-old APP-PS1 male and female mice (Hu et al., 2021). In addition to this intra-state diversity reported for the DAM signature, both the abundance and gene enrichment scores were found to be affected by the utilized mouse strain (Yang et al., 2021), further stressing the myriad of factors capable of affecting microglial heterogeneity.

Moreover, the DAM signature transcriptomically overlaps with the activated-response microglia (ARM), another microglial state identified in the cortex and hippocampus of 3, 6, 12 and 21-month-old APP^NL–G–F^ and C57BL/6 male and female mice, the latter which increases with age and pathology (Sala Frigerio et al., 2019). This signature seems to be strongly driven, however, by Aß as shown by the drastic increase of the hippocampal ARM population in 10/11-month-old *vs* 4-month-old APP-PS1 mice, and was also mildly increased in a tau pathology mouse model (Thy-Tau22 mice) [mouse model with G272V and P301S mutations in MAPT (Schindowski et al., 2006)] at 4 *vs* 10/11 months of age (Sierksma et al., 2020). Fibrillar Aß was further associated with an age-related progressive alteration of microglial signatures, reflected through changes in microglial Aß response proteins (MARPs), which partially overlap with the DAM signature and show impaired phagocytosis in two models of amyloid deposition: in 1, 3, 6 as well as 12 month-old APP-PS1 and APP^NL–G–F^ mice, both male and female (Sebastian Monasor et al., 2020). Other microglial states reported in AD pathology include the MGnD, which displays a down-regulation of homeostatic genes (e.g., *p2ry12, tmem119, fcrls*) and an up-regulation of genes such as *apoe, clec7a*, and *trem2*, linked to the phagocytosis of apoptotic neurons and dystrophic neurites in 24-month-old male and female APP-PS1 mice (Krasemann et al., 2017). It was also reported that the MGnD state presents an increased secretion of extracellular vesicles containing tau in 6-month-old male and female APP^NL–G–F^ mice injected with adeno-associated virus (AAV)-P301L-tau, highlighting this state’s potential to propagate tau (Clayton et al., 2021).

While partial or overlapping DAM signatures were uncovered in numerous mouse models of AD pathology, the particular DAM signature did not correlate with the lipid droplet-accumulating microglia (LDAM), a state associated with increased production of reactive oxygen species (ROS) and impaired phagocytosis, in the hippocampus of 20-month-old C57BL/6 and GRN-/- male mice, a model of frontotemporal dementia (Marschallinger et al., 2020). Recent studies have, however, revealed a conservation of the DAM signature in brains of patients with AD in comparison to healthy individuals. Indeed, using single-nucleus (sn) RNA-seq, Gerrits et al., identified two microglial profiles: AD1–composed of three subclusters enriched for some DAM genes (e.g., *LPL*, *ITGAX*, *SPP1*) (higher abundance found in patients with AD) and AD2–three regrouped microglial subclusters enriched notably in homeostatic genes (higher abundance in healthy control patients) (Gerrits et al., 2021). A reduced expression of P2RY12, a marker traditionally linked to homeostatic microglia and downregulated in mouse disease-associated microglial signatures [DAM (Keren-Shaul et al., 2017), MGnD (Krasemann et al., 2017)], and dark microglia (Bisht et al., 2016b) was observed in frontal and temporal cortices of human post-mortem AD brains compared to control individuals (Maeda et al., 2021). While some studies found an overlap between the mouse DAM and human microglial signatures in the post-mortem prefrontal cortex of patients with AD (Mathys et al., 2019), others have instead shown that this distinct population was not conserved. Indeed, human AD microglia (HAM) in the frontal and temporal cortex do not possess the same profile described in mouse models of AD pathology, but are present in another neurodegenerative condition (post-mortem brains samples of patients with multiple sclerosis) (Srinivasan et al., 2020). A lack of DAM in human was also reported by Sobue et al., in the precuneus of individuals with AD (Sobue et al., 2021). A summary of microglial states identified transcriptomically in AD pathology is provided in Table 2.

**TABLE 2:** Summary of microglial and astrocytic states observed in mouse models of AD pathology and human post-mortem brains of patients with AD.

State	Model	Technique	Age	Sex	Main finding	Main genes	References
**Microglia**							
Activated-response microglia (ARM)	APP^NL–G–F^ mice APP^swe^/PS1L166P and Thy-TAU22 mice	Single cell RNA sequencing Immunohistochemistry Bulk RNA sequencing	3, 6, 12 and 21-months 4, 10, 11 months	Male and female Male	Present during normal aging but increase with AD	Expression of genes associated with inflammation (*cst7*, *clec7a*, *itgax*), and major histocompatibility complex class II (*cd74*, *h2*-*Ab1*, *h2*-*Aa*, *ctsb, ctsd*)	Sala Frigerio et al., 2019 Sierksma et al., 2020
AD1	Human post-mortem (controls, AD)	Single-nucleus RNA sequencing	NA	NA	Higher abundance found in patients with AD	Increased expression of genes detected in DAM microglia including *ITGAX, LPL, GPNMB* and *SPP1*	Gerrits et al., 2021
AD2	Human post-mortem (controls, AD)	Single-nucleus RNA sequencing	NA	NA	Higher abundance found in control patients	Increased expression of homeostasis genes, (*CX3CR1* and *P2RY12*)	Gerrits et al., 2021
Disease-associated microglia (DAM)	5xFAD mice TREM2^+/–^5xFAD, TREM2^+/+^5xFAD, TREM2^–/–^ 5xFAD mice App^NL–G–F^ mice Tg2576, TgCRND8 mice CRND8 mice A/T/N mouse model (5xFADxPS19 mice injected with/without tau seeds) APP-PS1xTau P301L, APP-PS1 and Tau P301L mice APP751sl, ThyTau22 and P301S mice APP^NL–G–F^ mice, rTg4510 and human post-mortem APP-PS1 mice on C57BL/6, CAST, PWK and WSB strains	Single-cell RNA sequencing, immunofluorescence qPCR, immunofluorescence FACS, RNA sequencing RNA sequencing RNA sequencing Single-cell RNA sequencing Immunofluorescence, RNA sequencing, qPCR qPCR, flow cytometry RNA sequencing Single-cell RNA sequencing	1, 3, 6 and 8 months 7 months 9 months TgCRND8: 1.5, 3, 4.5, 6 and 10 months Tg2576: 3, 6, 9, 12 and 15 months 3, 6, 12 and 20 months 7 months 13 months 9–18 months APP751sl, 2–4 and 9–12 months ThyTau22 and P301S 7–8 months (mice) NA (human) 9 months	Male and female Male and female Male and female Male TgCRND8 and Female Tg2576 Male and female NA Female Male and female Male and female NA Female	Associated with a TREM2-dependent decrease of homeostatic genes and increase of disease signature genes	Downregulation of the purinergic receptors *p2ry12/p2ry13*, *cx3cr1*, and *tmem119*. Upregulation of AD risk factors (*apoe*, *ctsd*, *lpl*, *tyrobp* and *trem2*)	Keren-Shaul et al., 2017 Delizannis et al., 2021 Swartzlander et al., 2018 Rothman et al., 2018 McFarland et al., 2021 Lodder et al., 2021 Natunen et al., 2020 Romero-Molina et al., 2018 Sobue et al., 2021 Yang et al., 2021
							
Human AD microglia (HAM)	Human post-mortem (controls, AD)	Single cell/nucleus RNA sequencing	< 20 years old 64 ± 16 years	Male and female	Different profile in humans than in mouse models (DAM). Can be found in other neurodegenerative conditions	Downregulation of *MEF2C and* upregulation of *ABCA7, GPR141, PTK2B, SPI1* and *ZYX*	Srinivasan et al., 2020
Lipid droplet-accumulating microglia (LDAM)	C57BL/6 and GRN^–/–^ mice	RNA sequencing, immunohistochemistry, lipodomics, electron microscopy	18–20 months	Male	Associated with an increase in reactive oxidative species production and impaired phagocytosis	Expression of *slc33a1*, *snx17*, *vps35*, *cln3*, *npc2* and *grn*	Marschallinger et al., 2020
Microglial amyloid beta response proteins (MARP)	APP-PS1 and APP^NL–G–F^ mice	Mass spectrometry, immunofluorescence,	1, 3, 6 and 12 months	Male and female	Partially overlaps with the DAM signature (e.g., increase in CLEC7a, APOE) and show impaired phagocytosis	N/A	Sebastian Monasor et al., 2020
Neurodegenerative disease phenotype (MGnD)	APP-PS1 mice APP^NL–G–F^	Immunohistochemistry, RNA sequencing, qPCR Immunofluorescence, qPCR, FACS	9 and 24 months 6 months	Male and female Male and female	Associated with Aß-plaques and phagocytosis of apoptotic neurons, increased secretion of extracellular vesicles containing tau	Upregulation of *spp1, itgax, axl, lilrb4, clec7a, ccl2, csf1*, and *apoe*	Krasemann et al., 2017; Clayton et al., 2021
Senescent DAM	APP-PS1 mice and human post-mortem brains	Immunohistochemistry and gene expression analysis	4, 6, 10 and 12/13 months (mice) NA (human)	Male and female (mice) NA (human)	A substate of the DAM population, indicated by telomere shortening, increased beta-galactosidase activity and changes in gene expression associated with senescence	Upregulation of *cdkn1a, glb1*, and *serpine1*	Hu et al., 2021
Astrocytes							
Disease-associated astrocyte (DAA)	5xFAD mice Human post-mortem brains (controls, AD)	Single-nucleus RNA sequencing Single-nucleus chromatin accessibility and transcriptomic characterization	1.5–2, 4–5, 7–8, 10, 13–14 and 20 months 74–90 years old	Male and female Male and female	DAA were suggested to be Fos like-2 dependent	Upregulation of *serpina3n*, and *ctsb, apoe and clu*	Habib et al., 2020; Morabito et al., 2021;
Reactive astrocyte state	Human post-mortem brains (control, AD)	Cyclic multiplex fluorescent immunohistochemistry	76.7 ± 11.2 years	Male and female	Identified by increased expression of astrocytic markers GFAP and YKL-40.	N/A	Muñoz-Castro et al., 2022
							

Aß, amyloid beta; AD, Alzheimer’s disease; APP, amyloid precursor protein; FACS, fluorescence-activated cell sorting; GFAP, glial fibrillary acidic protein; NA, not available; PS1, presenilin 1; qPCR, quantitative polymerase chain reaction.

While the most commonly used models of AD pathology at this point continue to be non-primates [reviewed in Jankowsky and Zheng (2017)], there are notable gaps between these microglia and the ones studied in human post-mortem samples. The challenge in translating results across species could in part result from the difficulty in removing intact microglial cells from post-mortem brain samples and the artifacts associated with long post-mortem intervals (Srinivasan et al., 2020), as well as a lack of murine models fully replicating the intricate features of the disease (Boche and Gordon, 2021). Of note, non-human primate models of AD, such as the common marmoset, have shown incredible similarities to human Aß pathology (Latimer et al., 2019; Li et al., 2019). Nonetheless, non-primate models of AD pathology have provided invaluable insights into microglial heterogeneity that highlight the complexity of microglia as a glial and immune cell type. An area of increasing interest is the design of specific radiotracers to study inflammation within the CNS through positron emission tomography (PET) [reviewed by Narayanaswami et al. (2018)]. This non-invasive, *in vivo* technique has been used previously to investigate microglial activity in conjunction with the (neuro)inflammation marker, translocator protein 18-kDa (TSPO) expressed on the outer membrane of mitochondria (Ghadery et al., 2019; Zhang L. et al., 2021). It is important to note that TSPO is not specific to microglia, being also expressed by other CNS cells (e.g., astrocytes) (Zhang L. et al., 2021). In addition, TPSO’s expression was shown to vary between rodents and human, where under pro-inflammatory conditions (treatment with lipopolysaccharide), TSPO gene expression increased in primary microglial cells from postnatal day 0–5 C57BL/6 mice contrary to adult microglia isolated from the temporal lobe of patients with epilepsy (Owen et al., 2017). Although there are many shortcomings to microglial PET analysis, further advances could lead to the development of specific radiotracers to study *in vivo* microglial heterogeneity in humans.

## Astrocyte heterogeneity in Alzheimer’s disease

“Astrocyte reactivity” commonly refers to the plasticity or remodeling these cells undergo when responding to micro-environmental changes during pathological conditions (Escartin et al., 2021). Considered to represent a spectrum of varying states beginning in the developmental stages (Schitine et al., 2015; Clarke et al., 2021), astrocytes are often categorized based on their function and/or outcome on the brain (e.g., neuroprotective *vs* neurotoxic) (Liddelow et al., 2017; Miller, 2018; Matias et al., 2019; Westergard and Rothstein, 2020). The heterogenous nature of these glial cells ranges from varying morphology, ultrastructure to -omic signatures, to name a few. Micro-environmental changes, in both murine models of AD pathology and in human post-mortem brains of patients with AD, were also shown to drive this heterogeneity. For instance, astrocytes’ close interaction with AD hallmarks (e.g., dystrophic neurites, Aß plaques) can alter their morphology, notably resulting in hypertrophic and atrophic phenotypes (Zhou et al., 2019) as well as their gene and protein expression [e.g., GFAP, serpina3n, aquaporin 4 (AQP4), intercellular adhesion molecule 1 (ICAM-1)] (Akiyama et al., 1993; Orre et al., 2014; Habib et al., 2020; Spanos and Liddelow, 2020; Morabito et al., 2021).

### Morphological and ultrastructural heterogeneity of astrocytes

On a morphological aspect, astrocytes are distinguished by their numerous branches that can further ramify, giving them a star-like appearance in light microscopy [for a detailed review on protoplasmic astrocytes morphology, see Zhou et al. (2019)] (Şovrea and Boşca, 2013). Similar to microglial spatial heterogeneity, astrocytes from different brain regions (e.g., cortical layers I-VI, hippocampus) possess diverse morphologies associated with specific transcriptomic clusters (e.g., larger and more arborized cells in the hippocampus CA3 *vs* smaller ones in the cortical layers 2–4) (Batiuk et al., 2020). In AD pathology, numerous studies denoted a change in astrocytic morphology associated with atrophy, including in human post-mortem brains of late-Braak stages (Verkhratsky et al., 2019), and in mouse models of AD pathology (Olabarria et al., 2010; Yeh et al., 2011; Kulijewicz-Nawrot et al., 2012; Diniz et al., 2017). This atrophy was particularly evident in 3xTg male mice [Swedish mutation in APP, P301S mutation in MAPT and M146 mutation in PSEN1 (Oddo et al., 2003)], among the medial prefrontal cortex (mPFC) at 3, 9, 12 and 18 months of age (Kulijewicz-Nawrot et al., 2012), the dentate gyrus at 6, 12 and 18 months of age (Olabarria et al., 2010) and the entorhinal cortex at 1, 3, 6, 9 and 12 months of age (Yeh et al., 2011). In addition, intracerebroventricular injections of oligomeric Aß in the hippocampus CA1 *stratum radiatum* of 3-month-old Swiss male mice were associated with an atrophy of the astrocytic cytoskeleton resulting in a decreased number of processes and reduced cell area (Diniz et al., 2017).

Other studies have shown an increased cytoskeleton hypertrophy or increased complexity of astrocytes near Aß plaques using GFAP immunolabeling in the hippocampus of 6, 12, and 18-month-old TgF344 male and female rats [Swedish mutation in APP and humanized PSEN1 (Cohen et al., 2013)] (Mampay et al., 2020), in the dentate gyrus and hippocampus CA1 of 18-month-old 3xTg male mice (Olabarria et al., 2010) and in the cerebral cortex of 3, 6 and 12-month-old APP-PS1 mice (increase in volume and process length) (Li et al., 2020). A sex-specific increase in the abundance of hypertrophic astrocytes in 7–9 month-old APP-PS1 female compared to male mice was observed in the outer molecular layer of the dentate gyrus (Richetin et al., 2017). These differences highlight the significant impact of the micro-environment, notably AD hallmarks, alongside the effect of sex, age, and model, on the morphological differences observed across studies. As aforementioned, an atrophy and/or hypertrophy of astrocytes has been shown in AD pathology, however, the variety of regions, models, species, ages, and analytic techniques makes it challenging to correlate morphological heterogeneity in human AD cases. For instance, in the subiculum of human post-mortem brain samples of early- and late-onset AD compared to healthy individuals, 3D reconstruction of astrocytes using GFAP immunolabeling showed no morphological differences (volume, cell area) (Taipa et al., 2018).

As shown with microglial studies, EM was used by numerous research teams to investigate the ultrastructure and function of astrocytes in AD pathology. Indeed, Lin et al., demonstrated the presence of tau filaments within astrocytes in the spinal cord white matter of a JPNL3 [expressing the tau mutation P301L (Lewis et al., 2000)] mouse model of tauopathy (Lin et al., 2003). Others found astrocytic engulfment of dystrophic neurites near Aß plaques in the hippocampus of 6 and 12-month-old APP-PS1 male mice and in the medial temporal lobe of patients with AD, further clarifying the phagocytic role of astrocytes that appears conserved across species (Gomez-Arboledas et al., 2018). Astrocytic processes were also shown to pierce inside the amyloid core of a large mature plaque where they fragmented the latter, underlining the intimate relationship between these two elements (Wegiel et al., 2000). EM was also pivotal in shedding light on certain astrocytic dysfunctions in AD, including mitochondrial ultrastructural alterations (Baloyannis, 2019) and the autophagic functions *in vitro* of APOE3 and APOE4 astrocytic cells from the cerebral cortices of postnatal day 1–3 mice (Simonovitch et al., 2016). Changes in the expression of the water channel AQP4 between perivascular astrocytic end-feet and plaque-associated astrocytes was also uncovered in 5xFAD and tg-ArcSwe [Swedish and Artic mutation in APP (Lord et al., 2006)] mice using this imaging technique (Yang et al., 2011, 2017; Smith et al., 2019). Despite EM’s extensive applications, few studies have explored the ultrastructural diversity and heterogeneity of astrocytes in AD pathology using this approach. Therefore, the field would greatly benefit from in-depth EM studies investigating astrocytes, similar to what was uncovered in microglia from mouse model of AD pathology (i.e., dark microglia) (Bisht et al., 2016b) and in post-mortem brain samples of individuals with AD (El Hajj et al., 2019) and schizophrenia (i.e., dystrophic-like microglia) (Uranova et al., 2018).

### Heterogeneity of astrocytic molecular signatures in Alzheimer’s disease

Many recent efforts to uncover astrocytic heterogeneity have employed -omics techniques to characterize the varying astrocytic states observed in AD pathology (see Table 2 for a summary of astrocytic molecular signatures in AD). Like microglia, astrocytic regional differences can already be appreciated at steady-state, prior to dishomeostasis driven by micro-environmental changes (John Lin et al., 2017). Indeed, scRNA-seq investigations have identified 5 astrocytic subclusters heterogeneously dispersed in both the cortex and the hippocampus of 2-month-old C57BL/6 mice (Batiuk et al., 2020). For instance, cortical cluster 1, which associated with a mature astrocytic signature [high expression of *gfap* and angiotensinogen (*agt*)], was located in the subpial layer while cluster 5, an intermediate between mature and immature astrocytes (genes linked notably with mitosis and energy metabolism), was found in cortical layers 2/3 and 5 (Batiuk et al., 2020). With the objective of determining heterogeneity based on transcriptional signatures, astrocytes were studied using snRNA-seq in the hippocampus and cortex of 7-month-old 5xFAD male and female mice (Habib et al., 2020). In this study, 6 transcriptional states were distinguished by their uniquely up- and down-regulated genes, including the disease-associated cluster, which expressed genes associated with APP processing and Aß production (e.g., *serpina3n, ctsb*) and clearance (e.g., *apoe, clu*), as well as inflammatory signaling (Habib et al., 2020). In later studies, the disease-associated astrocytic signature was suggested to be dependent on Fos like-2 (FOSL2) (Morabito et al., 2021), a subunit of the activator protein 1 (AP-1) transcription factor (Lee et al., 2021).

As for microglial studies examining the translation of disease-associated states from mouse to human, conservation of the astrocytic disease-associated gene signature across species is still considered controversial in AD. A summary of the overlap between disease-associated astrocytes and other astrocytic states mentioned in this review is provided in Figure 1. For instance, Smith et al., uncovered 6 astrocytic subclusters, located in the entorhinal and somatosensory cortex from aged non-diseased control individuals (Braak 0-II) and patients with AD (Braak III-VI), using snRNA-seq. These subclusters were enriched for genes associated with diverse astrocytic roles and pathways from homeostatic functions (e.g., neurotransmitter uptake) to immune responses (Smith et al., 2022). The disease-associated signature was not attributed to a unique cluster, highlighting differences in astrocytic populations between species (Smith et al., 2022). Other astrocytic signatures found in the post-mortem human brain of patients with AD *vs* age-matched controls include a reactive astrocytic state, denoted notably by a high expression of the astrocytic proteins GFAP and chitinase 3-like 1 (chi3l1 or YKL-40), which was observed in the temporal association cortex using cyclic multiplex fluorescent immunohistochemistry combined with spectral clustering analysis (Muñoz-Castro et al., 2022). Human astrocytic heterogeneity in the post-mortem human AD brain was also described by Zhou et al. using snRNA-seq analysis in prefrontal cortex samples which showed a down-regulation of genes associated with the metabolic coordination between neurons and astrocytes [e.g., superoxide dismutase 2 (*sod2*), hypoxia inducible lipid droplet associated (*hilpda*), fatty acid binding protein 5 (*fabp5*)] (Zhou et al., 2020). While the aforementioned studies have helped to enlighten the heterogeneity of astrocytes in humans and mouse models of AD pathology, further investigation is required to fully unravel the puzzle behind the functions of the various astrocytic states observed in AD.

## Discussion

The evidence for the involvement of both diverse microglial and astrocytic states in the etiopathology of AD has been outlined above. *In situ*, microglia and astrocytes, as well as other cell types found throughout the CNS, exist in a pool of cellular signals, constantly exchanging information notably regarding the micro-environmental status (Fakhoury, 2018; Jha et al., 2019). Every step the field takes toward understanding these cellular and molecular interactions is another step closer to fully understanding the pathogenesis of AD. Within the broad categories of “microglia” and “astrocytes” lays even further complexity with recent studies demonstrating distinct states of both microglia (Bisht et al., 2016b; Keren-Shaul et al., 2017; Krasemann et al., 2017; Rangaraju et al., 2018; Hashemiaghdam and Mroczek, 2020; Nguyen et al., 2020; Xu et al., 2021; Yang et al., 2021) and astrocytes (Cunningham et al., 2019; Batiuk et al., 2020; Sofroniew, 2020). Some of the key questions that remain: How do these heterogeneous glial cells interact with each other? How do their interactions participate in the pathogenesis of AD?

The prominent role that both glial cells play in the pathogenesis of neurodegenerative diseases [through mechanisms such as blood-brain-barrier decay, extracellular matrix alterations, and production of ROS, for example (Serrano-Pozo et al., 2013; Acosta et al., 2017; Cragnolini et al., 2019; Gleichman and Carmichael, 2020; Liddelow et al., 2020)] also emphasizes the importance of studying inter-glial communication. This bidirectional communication was demonstrated to occur through released cytokines, metabolites, and neurotransmitters, among other mediators [reviewed in Jha et al. (2019)].

Because of the demonstrated roles of astrocytes and microglia in CNS inflammatory processes (Fakhoury, 2018), a comprehensive analysis of inflammatory signaling between glial cells reveals possible mechanisms for AD pathogenesis. Through the analysis of microglial inflammatory signaling molecules on astrocytes *via* a number of transgenic murine models, it has been demonstrated that the beneficial and/or harmful effects of astrocytes can be regulated by microglia and their subsequent cytokine secretions [tumor necrotic factor-alpha, complement component c1q and IL-1 alpha, for instance; (Liddelow et al., 2017; Shinozaki et al., 2017; Jha et al., 2019; Kim and Son, 2021)]. However, this relationship is not unidirectional, rather, the various microglial states and functions are heavily influenced by astrocytic signaling—particularly through plasminogen activator inhibitor-1 (Jeon et al., 2012), orosomucoid-2 (Jo et al., 2017), lipocalin-1 (Jha et al., 2015, 2019), pentraxin related protein-3 (Jeon et al., 2010), AQP4 (Smith et al., 2019) and trophic factors (Rocha et al., 2012; Norden et al., 2014) to name a few. For example, depletion of astrocytic lipid-binding protein, APOE, was shown to decrease tau-associated neurodegeneration and decrease microglial phagocytosis (Wang et al., 2021), and research is frequently emphasizing the role of APOE-TREM2 pathways as a means of microglial-astrocyte communication, particularly in modulating microglial homeostasis (Yeh et al., 2016; Krasemann et al., 2017; Taylor et al., 2020; Zhou et al., 2020; Mahan et al., 2022; Smith et al., 2022). Further, it has been demonstrated in 7-month-old APP/TTA (tetracycline transactivator) male mice that microglia-astrocyte communication through the complement system, specifically the complement C3, was involved in Aß pathology (Lian et al., 2016). Inhibition of this pathway, specifically *via* a signal transduction and activator of transcription-3 (STAT3) inhibitor, can mitigate tau pathology and inflammatory markers in the brain (excluding cerebellum) of PS19 tau transgenic mice (Litvinchuk et al., 2018). Another promising avenue of research pertain to toll-like receptors (TLRs) that many studies have implicated in the pathogenesis of AD (Liu et al., 2012; Li L. et al., 2021). Specifically, there is evidence that extracellular Aß and alpha-synuclein activate and upregulate TLR-2 and TLR-4, subsequently increasing a production of inflammatory mediators which interact with surrounding microglia and neuronal cells (Hughes et al., 2020; Li L. et al., 2021). Overall, TLRs, APOE, and C3 are molecules at the intersection of microglia-astrocyte communication which have demonstrated prominent roles in AD and are worth further investigation.

There is a substantial difficulty in deciphering the independent mechanisms of astrocytes and microglia in the pathogenesis of AD—making the investigation of their interactions and combined effects further complex. As described above, one promising methodology that is currently being utilized to investigate cellular heterogeneity and even to measure potential drug candidates for AD is multidimensional sc/sn-RNA-seq (Hammond et al., 2019; Batiuk et al., 2020; Olah et al., 2020; Xu et al., 2021; Smith et al., 2022). While a myriad of astrocytic and microglial states have been uncovered in recent years, various considerations must be taken into account when interpreting the transcriptomic heterogeneity of glial cells. Indeed, it is still unknown if the many microglial and astrocytic states observed are the results of AD pathology itself or technical artifacts. For instance, visualization of scRNA-seq and its interpretation can be distorted when using *t*-distributed stochastic neighbor embedding (*t*-SNE), which does not provide information on cellular heterogeneity based on neighboring cells, but rather on gene set. This difference can result in the visual misrepresentation of dense clusters when interpreting the data (Narayan et al., 2021). In addition, a myriad of technical and biological difficulties in scRNA-seq have been recently reported, from batch effect to incorrectly reported data and differing statistical approaches (Tung et al., 2017; Hicks et al., 2018; Squair et al., 2021), as well as artifacts caused by cell preparation (Nguyen et al., 2018). These variations highlight the challenges encountered when using the scRNA-seq technique to accurately study glial heterogeneity as well as the need to complement sequencing experiments with *in situ* techniques. In addition, with the expending popularity of this technique, various protocols were established (e.g., Smart-seq, Smart-seq2, MARS-seq) with differing results obtained in terms of gene detections and sensitivity, making it difficult to compare results across transcriptomic studies from different research groups (Ziegenhain et al., 2017). In isolated microglia, it has been shown that enzymatic digestion of the brain to generate single-cell samples affected their gene expression, underlining the sensitivity of this cell type to environmental changes and the need for careful processing (Marsh et al., 2022).

Moreover, further limitations pertaining to the varying states generated from sc- and snRNA-seq have to be taken into account. While snRNA-seq has gained popularity over single-cell due to its possible usage on frozen samples (Lake et al., 2016; Hu et al., 2017; Bakken et al., 2018; Thrupp et al., 2020), it remains controversial if this technique is suitable for glial cell investigation. Some studies have shown similar transcriptomic results in 2-month-old C57BL/6 male mice and in the superior frontal gyrus of human post-mortem brains when comparing fresh *vs* frozen samples as well as sc- *vs* sn- techniques (Gerrits et al., 2020). However, others have shown that snRNA-seq did not properly detect microglia in the temporal cortices of human brains (Thrupp et al., 2020), which would provide a possible explanation for the lack of translation regarding the mouse glial states (e.g., DAM) in human samples. Another reason behind the lack of gene conservation measured across species could arise from the cells themselves as differences between the transcriptomes of human and mouse astrocytes, for instance (e.g., responses to oxidative stress and hypoxia, metabolic differences), were reported (Li J. et al., 2021).

The abundant research described throughout this review, focusing on heterogeneity within the field of glia, does not paint a simple picture of isolated roles, singular functions, and straightforward interactions. Instead, with every new discovery attributing a discrete function to certain cytokines, lipoproteins, enzymes, or receptors, or with the imaging of a new functional state for each glial cell, the whole picture becomes further complex. The exact role of each cellular states is still in discovery, with their location, time-point, genes, and macro- and micro-environment all appearing as critical variables in determining cellular expression. As further research is conducted, the specialization of our analyses (e.g., pertaining to receptor expression, mitochondrial ultrastructure, cytoplasmic density, cytokine release, for example) of these cells will become increasingly important to fully understand the function and involvement in disease-states. Further, as techniques in the fields of imaging and -omics continue to develop, it is likely that the number of distinct categories will grow further as well. As described above, even within a particular glial state, such as DAM (Keren-Shaul et al., 2017; Rangaraju et al., 2018; Hu et al., 2021), there is immense diversity observed. Therefore, integrating different experimental approaches into mutual understanding is essential to the future of this field. With all this in mind, glial heterogeneity opens the idea that, while they may be categorized in simple terms, microglia and astrocytes account for a large number of distinct cell types and functional states and that these cellular states may act in accordance with each other but also, perhaps, in opposition. These various possibilities are important to keep in mind as these cellular states are investigated in health and in neurological disorders such as AD. As both the fields of microglia and astrocytes continue their respective progress, communication between these fields and concurrent research of glial communication is critical to providing discoveries in the search for therapeutic novelty in AD. Lastly, there is always a need for further examination in humans as those findings will inform preclinical experiments and help overcome translational barriers in AD research.

## Author contributions

M-KS-P, SL, and JV wrote the manuscript. M-KS-P and M-ÈT conceptualized and edited the review. M-KS-P designed the figure. All authors approved the submitted manuscript.
